# Antibodies Elicited by an NS1-Based Vaccine Protect Mice against Zika Virus

**DOI:** 10.1128/mBio.02861-18

**Published:** 2019-04-02

**Authors:** Mark J. Bailey, Felix Broecker, James Duehr, Fortuna Arumemi, Florian Krammer, Peter Palese, Gene S. Tan

**Affiliations:** a Department of Microbiology, Icahn School of Medicine at Mount Sinai, New York, New York, USA; b Graduate School of Biomedical Sciences, Icahn School of Medicine at Mount Sinai, New York, New York, USA; c Department of Medicine, Icahn School of Medicine at Mount Sinai, New York, New York, USA; d Infectious Diseases, The J. Craig Venter Institute, La Jolla, California, USA; e Department of Medicine, University of California San Diego, La Jolla, California, USA; Johns Hopkins Bloomberg School of Public Health

**Keywords:** Fc-mediated responses, NS1, Zika virus, antibody-dependent enhancement of disease, flavivirus, nonneutralizing antibodies, vaccine

## Abstract

Zika virus is a mosquito-borne flavivirus which can cause severe disease in humans, including microcephaly and other congenital malformations in newborns and Guillain-Barré syndrome in adults. There are currently no approved prophylactics or therapeutics for Zika virus; the development of a safe and effective vaccine is an urgent priority. Preclinical studies suggest that the envelope glycoprotein can elicit potently neutralizing antibodies. However, such antibodies are implicated in the phenomenon of antibody-dependent enhancement of disease. We have previously shown that monoclonal antibodies targeting the Zika virus nonstructural NS1 protein are protective without inducing antibody-dependent enhancement of disease. Here, we investigated whether the NS1 protein itself is a viable vaccine target. Wild-type mice were vaccinated with an NS1-expressing DNA plasmid followed by two adjuvanted protein boosters, which elicited high antibody titers. Passive transfer of the immune sera was able to significantly protect STAT2 knockout mice against lethal challenge by Zika virus. In addition, long-lasting NS1-specific IgG responses were detected in serum samples from patients in either the acute or the convalescent phase of Zika virus infection. These NS1-specific antibodies were able to functionally engage Fcγ receptors. In contrast, envelope-specific antibodies did not activate Fc-mediated effector functions on infected cells. Our data suggest that the Zika virus NS1 protein, which is expressed on infected cells, is critical for Fc-dependent cell-mediated immunity. The present study demonstrates that the Zika virus NS1 protein is highly immunogenic and can elicit protective antibodies, underscoring its potential for an effective Zika virus vaccine.

## Abstract (precis)

Zika virus is a global public health threat that causes microcephaly and congenital malformations in newborns and Guillain-Barré syndrome in adults. Currently, no vaccines or treatments are available. While antibodies targeting the envelope glycoprotein can neutralize virus, they carry the risk of antibody-dependent enhancement of disease (ADE). In contrast, antibodies generated against the NS1 protein can be protective without eliciting ADE. The present study demonstrates the effectiveness of an NS1-based vaccine in eliciting high titers of protective antibodies against Zika virus disease in a mouse model. Sera generated by this vaccine can elicit Fc-mediated effector functions against Zika virus-infected cells. Lastly, we provide human data suggesting that the antibody response against the Zika virus NS1 protein is long-lasting and functionally active. Overall, our work will inform the development of a safe and effective Zika virus vaccine.

## INTRODUCTION

Zika virus (ZIKV), a flavivirus related to dengue virus (DENV), caused an epidemic that spread rapidly across the globe in the past decade (1). ZIKV infection can cause severe disease in humans, including microcephaly in newborns and Guillain-Barré syndrome in adults (2–4). Although primarily spread by infected *Aedes* species mosquitoes, ZIKV can also be transmitted sexually or from mother to fetus (5, 6). Ongoing transmission in the Americas and India suggests that ZIKV is now endemic, and much of the world’s population is at continued risk of infection (7, 8). Due to the rapid spread of ZIKV and the particularly severe disease exhibited in developing human fetuses, effective vaccines and treatments are critically needed.

A number of studies of mice and nonhuman primates have shown the efficacy of multiple vaccine platforms (9). DNA, mRNA, adenovirus, and purified inactivated virus platforms all have shown promising results in both preclinical and phase I studies (10–14). Many of these vaccines are designed to protect against ZIKV infection by eliciting neutralizing antibodies that target the surface envelope, glycoprotein E. These envelope-specific antibodies can be potently neutralizing and provide sterilizing immunity (15, 16). However, an ongoing concern in the field of flavivirus vaccinology, and in dengue virus in particular, is the potential development of antibody-dependent enhancement (ADE) of disease (17). ADE occurs when antibodies bound to virions fail to neutralize the virus but facilitate virion internalization via the Fc receptors of innate immune cells. Increased viral internalization and subsequent replication leads to more-severe disease outcomes. At present, there is no human epidemiologic evidence that prior immunity to ZIKV enhances dengue disease or *vice versa*. However, *in vitro* and *in vivo* evidence suggests that enhancement of ZIKV or dengue virus can occur in experimental settings (18, 19). As such, a vaccine approach targeting nonenvelope viral proteins would minimize the potential for ADE of disease.

The immune response to acute flavivirus infection targets not only the E protein but also the nonstructural proteins, including NS1. The flaviviral NS1 protein has been implicated in immune evasion and viral replication and has both intracellular and extracellular functions (20). Intracellularly, the NS1 protein localizes to sites of viral RNA synthesis and is critical for genome replication (21). The NS1 protein is also trafficked to the plasma membrane, where it binds the surface of infected cells by a putative glycosylphosphatidylinositol linker. The secreted form exists as a hexamer and accumulates to high levels in sera and tissues. The extracellular form of the NS1 protein is highly antigenic and is thought to modulate the humoral immune response. The extracellular form of the dengue virus NS1 protein can also activate complement pathways, potentially leading to vascular leakage (22). In the context of ZIKV, the NS1 protein contributes to evasion of the host antiviral response and has been found to enhance the uptake of virus by mosquitoes (23, 24). A potent immune response to the NS1 protein may have multifaceted beneficial effects, including decreased transmission by halting the urban transmission cycle as well as reducing disease burden in humans by clearance of virally infected cells (25). Antibodies to the NS1 protein do not provide sterilizing immunity, as they are nonneutralizing. However, NS1-specific antibodies are known to activate Fc-mediated effector functions, such as antibody-dependent cell-mediated cytotoxicity (ADCC), antibody-dependent cell-mediated phagocytosis (ADCP), and antibody-dependent complement-mediated lysis (26–29). Additionally, recent studies suggest that the antibody response toward the ZIKV NS1 protein is highly specific and can be used for diagnostic purposes (30, 31). Work by our group has shown that human antibodies that target the ZIKV NS1 protein can provide protection in mice against lethal challenge by ZIKV in an Fc-dependent manner (32). We have shown a decrease in viral titer as well as reduction in morbidity and mortality in infected mice to which human anti-NS1 antibodies were passively transferred. Therefore, NS1 may prove a key component of an effective ZIKV vaccine.

The NS1 protein of flaviviruses was considered a potential component in vaccine preparations. Vaccination with yellow fever virus NS1 protein prevented encephalitis in mice and lethality in macaques upon viral challenge (33, 34). More recently, vaccination with the dengue virus NS1 protein prevented vascular leakage and disruption of endothelial barriers in mice (35). However, the dengue virus NS1 protein may also induce auto-antibodies that cross-react with host proteins present on endothelial cells and platelets, which may result in endothelial damage (36–39). These phenomena, however, have not yet been reported for antibodies targeting the Zika NS1 protein. Today, a few groups have studied the role that NS1-mediated immunity may play in protection against ZIKV. Brault et al. have shown that a ZIKV NS1 protein in a modified vaccinia virus Ankara vector protects mice from intracranial viral challenge (40). Two additional groups have combined NS1 with premembrane/membrane (prM/M) and E proteins and showed increased protection provided by NS1-prM/M-E compared to that provided by prM/M-E alone (41, 42). Notably, since none of these studies included passive-transfer experiments, it is unclear whether the antibody or cell-mediated immune response contributed most to protection against disease.

Here, we show that a vaccination regimen consisting of a DNA prime and two NS1 protein boosts elicited high titers of antibodies to the ZIKV NS1 protein in wild-type mice. We found that passive transfer of sera was sufficient to protect STAT2^−/−^ mice from lethal challenge, which suggests that the antibody-mediated immune response is critical to protect against disease. Sera from vaccinated mice engaged the Fcγ receptor (FcγR) in an *in vitro* Fc-FcγR reporter assay. We next determined that NS1-mediated immunity is robust and long-lasting in humans by analyzing serum samples from acute- and convalescent-phase ZIKV-infected patients. These antibodies generated against NS1 by natural viral infection are functionally active as measured by the same reporter assay. Notably, we show that while polyclonal cross-reactive envelope antibodies elicited the Fc-dependent ADE of infection *in vitro*, these cross-reactive antibodies did not activate Fc-FcγR effector functions against ZIKV-infected cells. Our current findings suggest that the NS1-specific antibody response allows for robust Fc-dependent cell-mediated immunity, which has broad implications in the design of effective flavivirus vaccines.

## RESULTS

### Vaccination with a DNA plasmid and NS1 protein elicits high titers of anti-NS1 IgG in mice.

**FIG 1:**
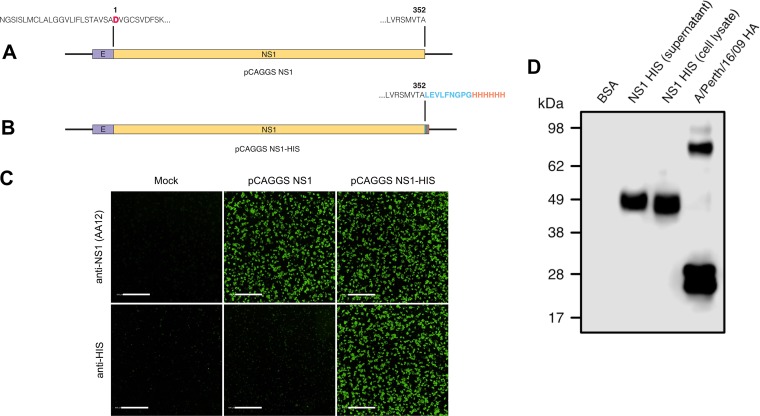
Generation of expression plasmids encoding ZIKV NS1. (A) Human-codon-optimized NS1 of ZIKV PRVABC59 was subcloned into a mammalian expression vector, pCAGGS, which includes the last 24 amino acids of the envelope protein at the amino terminus followed by the NS1 coding region (pCAGGS NS1). Of note, the first amino acid of the NS1 coding region is indicated by a bold, red aspartic acid residue. (B) A second version (pCAGGS NS1-His) also encodes ZIKV PRVABC59 NS1, followed by a PreScission Protease cleavage site (LEVLFNGPG; blue region) and a hexahistidine motif (HHHHHH; orange region) at the carboxy terminus. (C) HEK 293T cells were transfected with pCAGGS NS1 or pCAGGS NS1-His or not transfected (mock). At 24 h posttransfection, the cells were fixed with 0.5% paraformaldehyde, and the surface expression of NS1 was detected using an anti-ZIKV NS1 monoclonal antibody, AA12, or a polyclonal anti-histidine antibody. Secondary antibodies conjugated to Alexa Fluor 488 were used to visualize binding using a Celigo imaging cytometer. The scale bars are equal to 500 μm. (D) HEK 293F cells were transfected with pCAGGS NS1-His, and 4 days posttransfection, soluble NS1 from the supernatant and cell lysates was collected and purified over an NI-NTA column. Soluble NS1 proteins from the supernatant and lysates were resolved in an SDS-PAGE gel and detected using a polyclonal anti-histidine antibody in a Western blot assay. BSA was used as a negative control, and a His-tagged soluble hemagglutinin of A/Perth/16/09 (H3N2) was used as a positive control.

**FIG 2:**
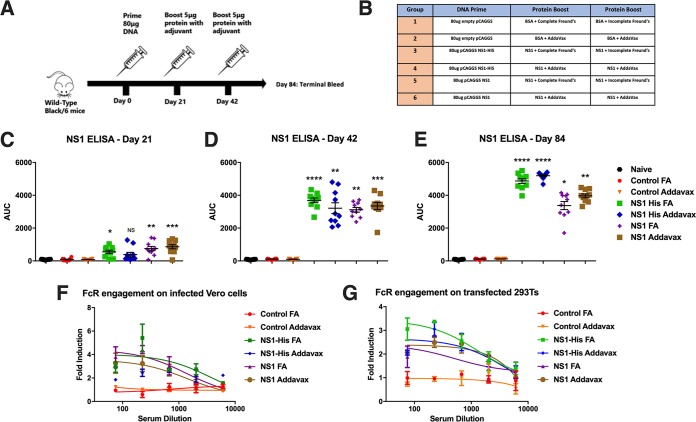
Zika virus NS1 vaccine induces a robust and functional antibody response in mice. (A) Schematic outlining the vaccination strategy, where mice were prime immunized with 80 µg of the pCAGGS NS1 DNA plasmid via electroporation, followed by two booster immunizations of soluble NS1 proteins. All vaccinations were administered intramuscularly. (B) Groups of mice vaccinated with each adjuvant used for the protein components. (C to E) The antibody response to NS1 (PRVABC59 ZIKV) was measured by ELISA. Each data point denotes an individual animal, while each color represents one group of mice. The time points are after the DNA priming at day 21, after the protein boost at day 42, or at day 84 (sera from the terminal bleed). ELISA data were run in duplicate and are shown as areas under the curve (AUCs). A nonparametric multiple-comparison Kruskal-Wallis test was used to determine statistical significance at each time point. Asterisks indicates statistical significance of a group (*, *P* < 0.05; **, *P* < 0.01; ***, *P* < 0.001; ****, *P* < 0.0001) in a comparison with naive serum. No significance was observed between control groups and the naive group. FA, Freund's adjuvant. (F and G) To examine the ability of NS1-specific antibodies to activate Fc-mediated effector functions, Vero cells were infected with PRVABC59 ZIKV or HEK 293T cells were transfected with an NS1 expression plasmid (pCAGGS-NS1). Infected Vero cells or transfected HEK 293T cells were used as targets for measuring antibody-mediated effector functions with a genetically modified Jurkat cell line expressing murine FcRγ IV with an inducible luciferase reporter gene. Fold induction was measured in relative light units and calculated by subtracting background signal from wells without effector cells and then dividing wells with sera by wells with no sera added. All sera were tested at a starting dilution of 1:75 and were serially diluted 3-fold in duplicate. A nonlinear regression best-fit curve was generated for each data set using GraphPad Prism 6. Error bars represent SEM.

Two ZIKV vaccine constructs were generated by introducing human codon-optimized sequences encoding the full-length NS1 protein from the Asian-lineage ZIKV PRVABC59 strain into a pCAGGS mammalian expression vector. The first construct, pCAGGS NS1, encodes the last 24 amino acids of the ZIKV envelope protein at the amino terminus, allowing for proper folding and anchoring of the NS1 protein to the lipid bilayer (43) followed by the complete coding region of the NS1 protein of ZIKV PRVABC59 (an aspartic acid marks the first residue of the NS1) (Fig. 1A). We also designed an expression plasmid designed to produce soluble NS1 protein. The pCAGGS NS1 expression plasmid was modified by adding a PreScission Protease cleavage site and a hexahistidine motif at the carboxy terminus, and the resulting plasmid was named pCAGGS NS1-His (Fig. 1B). To determine if these plasmids generated properly folded ZIKV NS1 protein, we transfected HEK 293T cells with pCAGGS NS1 or pCAGGS NS1-His. Mock-transfected cells served as a negative control. At 24 h posttransfection, expression of ZIKV NS1 on HEK 293T cells was confirmed using immunofluorescence by a human monoclonal antibody, AA12, or a polyclonal antihistidine antibody (32) (Fig. 1C). As expected, an antihistidine antibody detected only pCAGGS NS1-His. Mock-transfected cells were not detected by AA12 or polyclonal antihistidine antibody. Using the pCAGGS NS1-His plasmid, we expressed the NS1 protein in human embryonic kidney (HEK) Expi293 cells and purified the protein using a nickel-nitrilotriacetic acid (Ni-NTA) resin. Purified soluble NS1 protein was resolved by sodium dodecyl sulfate-polyacrylamide gel electrophoresis (SDS-PAGE) under both denaturing and reducing conditions. Western blot analysis using a polyclonal anti-His antibody demonstrated that the soluble His-tagged NS1 proteins purified from both the cell culture supernatant and lysates resolved at approximately 50 kDa as monomers (Fig. 1D). A soluble histidine-tagged hemagglutinin of influenza A virus strain A/Perth/16/09 (H3N2) was used as a positive control, while bovine serum albumin (BSA) served as a negative control. We then vaccinated groups of 10 mice as outlined by the vaccination strategy in Fig. 2A and B. At day 0, wild-type C57BL/6 mice were primed with either 80 μg of pCAGGS NS1-His or pCAGGS NS1 via intramuscular electroporation. Next, mice were immunized intramuscularly with 5 μg of adjuvanted NS1 protein at days 21 and 42. Mice receiving soluble NS1 protein with Freund’s adjuvant received complete adjuvant at day 21 and incomplete adjuvant at day 42. Mice receiving soluble NS1 protein with AddaVax received the same adjuvanted protein on both days 21 and 42. As a control, mice were vaccinated with an empty pCAGGS plasmid and boosted twice with BSA supplemented with either Freund’s adjuvant or AddaVax. Prior to administration of each vaccine component, serum samples were obtained by facial vein puncture. At day 84, the mice were anesthetized and terminally bled by cardiac puncture, and sera were collected for further analysis and passive-transfer studies.

An NS1-specific enzyme-linked immunosorbent assay (ELISA) was performed, and all NS1-vaccinated mice demonstrated a robust reactive antibody response after the DNA prime immunization followed by two protein boosts (Fig. 2C to E). Significant differences from the naive group were observed in all vaccine groups on day 42 and day 84. On day 21, there was no significant difference between the NS1-His AddaVax group and the naive group. However, this group had sufficiently high titers by days 42 and 84. No differences were observed between the naive group and either of the two control groups. Additionally, no differences were observed within each vaccination group. A trend toward higher titers in the groups vaccinated with the His-tagged construct was observed by day 84. This is likely due to elicited anti-His antibodies recognizing His-tagged NS1 proteins used in our ELISAs.

Immunofluorescence studies demonstrated reactivity to Vero cells infected with the Asian-lineage ZIKV PRVABC59 in all treatment groups (see Fig. S1 in the supplemental material). As the NS1 protein is not present on the Zika virion itself but is expressed on the surfaces of infected cells, NS1-mediated immunity is unlikely to be sterilizing. Rather, NS1-specific antibodies are likely to protect via antibody-dependent cell-mediated effector functions. To determine whether sera from vaccinated mice are functionally active against infected cells, we tested the ability of the NS1-vaccinated mouse sera to engage FcγRs. We used a well-established *in vitro* assay that we previously used to assess the functional activities of monoclonal antibodies targeting the influenza virus hemagglutinin and the Zika virus NS1 protein (32, 44). In this assay, engagement of the murine FcγR IV expressed on genetically modified effector (Jurkat) cells results in a quantifiable luminescent signal. We infected Vero cells with the ZIKV PRVABC59 strain and added pooled (*n* = 10) sera from vaccinated mice. Consistently with the ELISA results, we found that sera from all NS1-vaccinated groups induced effector functions on ZIKV PRVABC59-infected cells (Fig. 2F), while sera from the control group were unable to engage FcγRs. To confirm that Fc-mediated effector functions are NS1 specific, we transfected 293T cells with the pCAGGS NS1 plasmid. As with what we observed with infected cells, we found that all NS1-vaccinated groups induced effector functions but that the control groups did not (Fig. 2G), indicating that Fc-mediated activity was indeed NS1 specific.

10.1128/mBio.02861-18.1 FIG S1 Immunofluorescence of pooled mouse serum. Download FIG S1, DOCX file, 0.2 MB. Copyright © 2019 Bailey et al. 2019 Bailey et al. This content is distributed under the terms of the Creative Commons Attribution 4.0 International license.

### Passive transfer of immune sera protects STAT2^–/–^ mice from lethal challenge.

**FIG 3:**
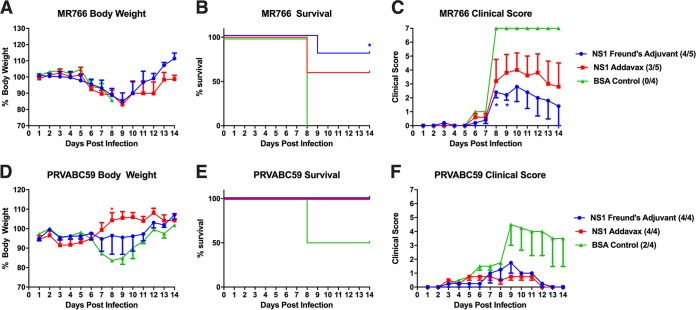
Passive transfer of serum from vaccinated mice protects against lethal challenge. (A to C) Groups of 4 to 5 male and female B6.129-Stat2^−/−^ mice were injected intraperitoneally with 200 μl of pooled serum before a challenge with 10 LD_50_s (158 PFU per mouse) of MR766 ZIKV intradermally. (D to F) Groups of 4 male and female B6.129-Stat2^−/−^ mice were injected intraperitoneally with 200 μl of pooled serum before a challenge with 1,000 PFU of PRVABC59 ZIKV intradermally. Weight loss was monitored daily. Clinical scoring was conducted using the predefined criteria, with a maximum possible score of 7: impact on walking (1), unresponsiveness (1), left hind leg paralyzed (1), right hind leg paralyzed (1), left front leg paralyzed (1), and right front leg paralyzed (1). Deceased animals were awarded a score of 7. The ratios in the figures indicate the number of animals that survived challenge over the total number of animals per group. The Mantel-Cox and Gehan-Breslow-Wilcoxon tests were used to analyze statistical significance of survival between two groups. A multiple *t* test and the Holm-Šidák method were used to determine statistical significance at each time point for the weight curve and the clinical score. Asterisks indicate statistical significance of a group (*, *P* < 0.05) in a comparison with mice vaccinated with BSA.

To determine whether antibodies elicited by our vaccine regimen are protective against ZIKV, we passively transferred 200 μl of pooled sera intraperitoneally into STAT2^−/−^ mice, which are permissive to ZIKV infection and can display clinical signs of disease (45). Two hours after administration of sera, the mice were challenged intradermally with 10 50% lethal doses (LD_50_) of the African-lineage ZIKV MR766 strain. Mice were monitored daily for weight loss and scored for signs of disease, including difficulty walking, limb paralysis, and unresponsiveness. Animals exhibiting a clinical score of 5 or higher were euthanized and scored as succumbing to disease. Sera from mice given an NS1 booster and Freund’s adjuvant provided the highest degree of protection, with 80% of the mice surviving the challenge, compared to 60% in the AddaVax group and 0% in the BSA control group (Fig. 3A to C). We next tested protection against the homologous Asian-lineage strain PRVABC59, which is more closely related to contemporary strains of ZIKV. We administered 1,000 PFU of the PRVABC59 virus, as a proper LD_50_ could not be administered due to a lack of virulence at the highest doses tested. We found that 100% of mice given sera from NS1-vaccinated mice survived the challenge with PRVABC59, compared to 50% of mice treated with control sera (Fig. 3D to F). As with the results of the MR766 challenge study, all mice displayed clinical signs of infection. We observed no differences in lethality between male or female mice. Though it is well established that ZIKV PRVABC59 displays less pathogenicity than MR766 in mice, we were still able to detect significant differences in weight loss between the NS1-vaccinated mice and control mice (45).

### NS1-mediated immunity is long-lasting in humans and mediates Fc effector functions.

**FIG 4:**
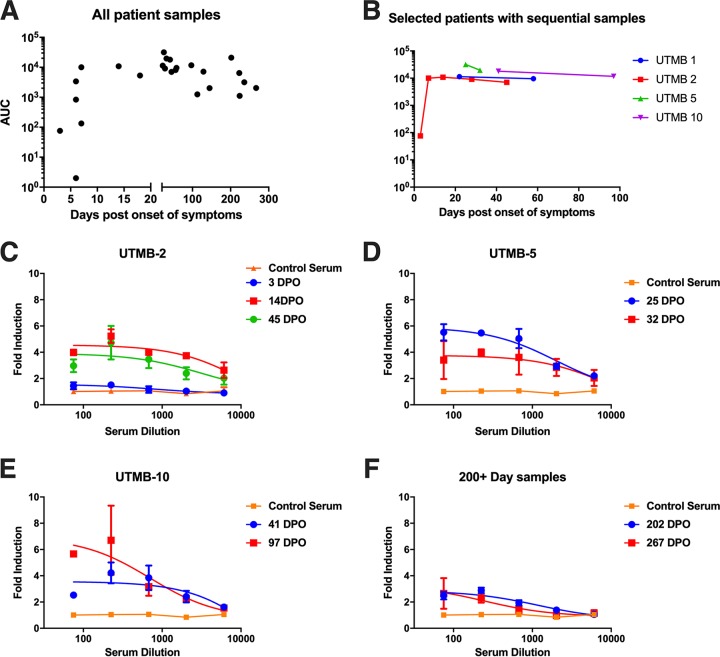
NS1-specific antibodies are long-lived and functional in ZIKV infected humans. ELISA data of individual human sera against recombinant NS1 protein from PRVABC59 ZIKV. ELISA data were run in duplicate, and values represent AUCs. (B) ELISA data of select human samples with repeat blood draws. Each color represents an individual patient. UTMB, patient designation. (C to F). To test the ability of human sera to activate Fc-mediated effector functions, Vero cells were infected with PRVABC59 ZIKV and an Fc-FcγR reporter assay was performed as previously shown. The legend includes the patient identifier number and the number of days postonset of symptoms (DPO). All sera were tested at a starting dilution of 1:75 and were serially diluted 3-fold in duplicate. A nonlinear regression best-fit curve was generated for each data set using GraphPad Prism 6. Error bars represent SEM.

**FIG 5:**
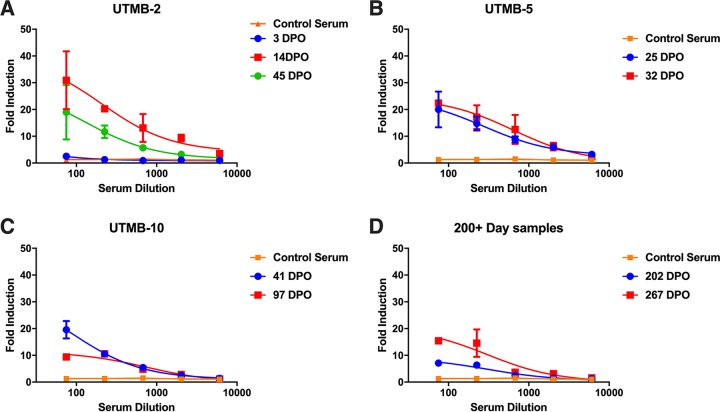
Human sera can engage FcγRs when targeting NS1-transfected cells. (A to D) To examine the ability of human sera to activate NS1-specific Fc-mediated effector functions, HEK 293T cells were transfected with an NS1 (pCAGGS-NS1) expression plasmid. Each color represents an individual sample; the titles includes the patient identifier, and the keys include numbers of days postonset (DPO) of symptoms. A surrogate *in vitro* reporter assay for measuring Fc-FcγR interactions was performed as previously shown (Fig. 4) (32). All sera were tested at a starting dilution of 1:75 and were serially diluted 3-fold in duplicate. A nonlinear regression best-fit curve was generated for each data set using GraphPad Prism 6. Error bars represent SEM.

The neutralizing activity of envelope-specific antibodies elicited during ZIKV infection is well documented and characterized (19, 46, 47). However, there are a paucity of data on the duration and mechanisms of action of NS1-specific antibodies in humans infected by ZIKV or other flaviviruses. To determine whether NS1-mediated immunity is relevant and long-lived in humans, serum samples were obtained from patients infected by ZIKV. These samples were taken from patients ranging from acutely ill to fully recovered, from 3 to 267 days postonset of symptoms (Tables S1 and S2). The reactivity of these serum samples to NS1 protein was determined by ELISA. We found that NS1-specific antibodies became detectable at approximately day 10 postonset of symptoms and remained elevated throughout day 267, with minimal waning over time (Fig. 4A). We also obtained serum samples from the same individuals at multiple time points to represent a longitudinal response. In these matched samples, we found that the NS1 response waned slightly over time but did not return to baseline levels (Fig. 4B). Next, we determined whether serum samples from these individuals were able to elicit Fc-FcγR-mediated effector functions. Using *in vitro* assays, we found measurable correlations between reactivity to NS1 and the ability to engage FcγR (Fig. 4C to F). For instance, patient UTMB-2 had a low antibody titer to NS1 at day 3 postinfection and likewise did not show effector function activity on ZIKV PRVABC59-infected cells at that time point. However, at days 14 and 45 postinfection, both the patient’s sera were reactive to NS1 by ELISA and functionally active, as measured by the ADCC reporter assay. Additionally, we tested two samples taken later than day 200 postinfection and found that these sera were still able to induce effector functions. Next, we determined whether antibodies to NS1 specifically contributed to the Fc-FcγR-mediated immunity by transfecting HEK 293T cells with a plasmid expressing NS1 and using the same ADCC reporter assay. Individuals who had a positive antibody response to infected Vero cells were found to also react with transfected HEK 293T cells (Fig. 5A to D). From these data, we conclude that the NS1 response elicited by natural ZIKV infection is long-lasting and contributes to Fc-mediated immunity in humans.

10.1128/mBio.02861-18.2 TABLE S1 Serum samples obtained from Zika virus-infected patients. Download Table S1, DOCX file, 0.01 MB. Copyright © 2019 Bailey et al. 2019 Bailey et al. This content is distributed under the terms of the Creative Commons Attribution 4.0 International license.

10.1128/mBio.02861-18.3 TABLE S2 Serum samples obtained from Zika virus-infected patients. Download Table S2, DOCX file, 0.01 MB. Copyright © 2019 Bailey et al. 2019 Bailey et al. This content is distributed under the terms of the Creative Commons Attribution 4.0 International license.

### Cross-reactive antibodies against the envelope protein do not elicit FcγR effector functions in humans.

**FIG 6:**
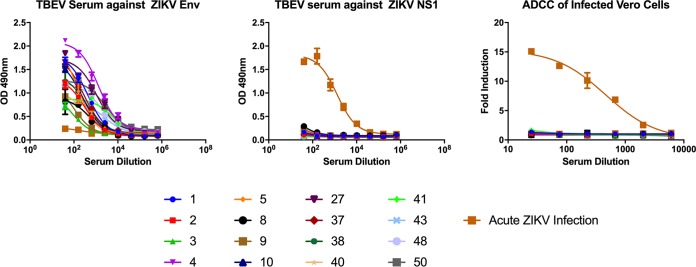
Cross-reactive envelope-specific antibodies do not elicit Fc-mediated responses. Sera from TBEV vaccinated patients were analyzed for binding to ZIKV proteins and the ability to elicit Fc-mediated effector functions. Sera were obtained through a screening of TBEV vaccinee samples as described previously in the work of Duehr et al. (49). ELISAs were performed on recombinant ZIKV envelope protein (A) or recombinant NS1 protein from PRVABC59 ZIKV (B). All sera were tested at a starting dilution of 1:40 and were serially diluted 4-fold in duplicate. “Acute ZIKV Infection” indicates serum from a patient with an acute infection confirmed by reverse transcription-PCR. (C) Vero cells were infected with PRVABC59 ZIKV, and a surrogate *in vitro* reporter assay was performed to measure Fc-FcγR interactions. All sera were tested at a starting dilution of 1:25 and were serially diluted 3-fold in duplicate.

Though a significant number of antibodies are generated against the NS1 protein, a larger portion of the antibody response is directed against the ZIKV envelope protein. Envelope-specific antibodies predominantly contribute to a potent neutralizing response and provide sterilizing immunity. Cross-reactive envelope-specific antibodies, however, are also known to be potent mediators of antibody-dependent enhancement (ADE) of disease (15). These antibodies are known to bind conserved epitopes near the fusion loop of the envelope glycoprotein and can bind divergent flaviviruses (48). Notably, in Duehr et al., 28 of 50 serum samples from tick-borne encephalitis virus (TBEV)-vaccinated individuals bound to recombinant ZIKV envelope protein by ELISA, while 36 of 50 serum samples had enhanced ZIKV infectivity *in vitro* (49). Since ADE of infection is Fc mediated, we sought to determine whether these same cross-reactive antibodies are able to elicit potentially beneficial Fc-mediated effector functions *in vitro* on infected cells. To answer this question, we analyzed a set of serum samples from individuals vaccinated against TBEV, a member of the flavivirus family (49). Though the amino acid sequences of the TBEV and the ZIKV envelope proteins are divergent, exhibiting approximately 40% identity at the amino acid level (49), cross-reactive antibodies against conserved epitopes near the fusion loop of domain II of the envelope protein are often generated (15). Sixteen of the highest ELISA- and ADE-reactive serum samples from the work of Duehr et al. were analyzed for binding to the recombinant ZIKV E protein by ELISA, and all showed a positive response (Fig. 6A). As a control, we used sera from an acute ZIKV infection known to have a strong NS1-specific response with low reactivity to recombinant ZIKV E (32). We next confirmed that the vaccinated samples did not have antibodies targeting the ZIKV NS1 protein. The TBEV vaccine, which was used to vaccinate the human subjects, uses inactivated TBEV virus. As this vaccine does not contain NS1, serum samples from TBEV patients did not react with ZIKV NS1, while the positive control, serum from an acutely infected individual, did react (Fig. 6B). We then tested whether these serum samples can elicit Fc-mediated effector functions in ZIKV PRVABC59-infected Vero cells. We found that out of the 16 TBEV-vaccinated serum samples tested, none were able to elicit Fc-mediated effector activity on ZIKV-infected cells but that sera from an individual acutely infected with ZIKV did (Fig. 6C). Our data suggest that while cross-reactive envelope-specific antibodies elicited by TBEV vaccination might cause ADE of infection to occur *in vitro,* they do not induce Fc-mediated effector functions on infected cells. We hypothesize that a strong NS1 antibody response is important for the clearance of ZIKV-infected cells via Fc-dependent cell-mediated activity. Conversely, due to the low levels of the envelope glycoprotein expressed at the surfaces of infected cells, cross-reactive E antibodies are unable to target these cells for Fc-mediated clearance.

## DISCUSSION

Several ZIKV vaccines are currently under various phases of development (10–14, 50–52). The aim of most of these candidate vaccines is to elicit potent naturalizing antibody responses using the envelope glycoprotein E as the major target antigen. ZIKV E-specific antibodies can provide sterilizing immunity, and characterization of a number of neutralizing monoclonal antibodies targeting the E protein revealed high neutralizing activity with half-maximal inhibitory concentrations in the nanogram-per-milliliter level (19, 46, 47, 53, 54). However, the ZIKV NS1 protein is an equally viable target for a vaccine. Previous studies have demonstrated antibodies against other flavivirus NS1 proteins from yellow fever virus (YFV), West Nile virus (WNV), or DENV can limit or prevent flavivirus disease (26–28, 33–35). Additionally, vaccines that elicit NS1-specific antibodies do not cause antibody-dependent enhancement of disease, as these antibodies do not bind to the virion itself. This is pertinent, as the vaccine Dengvaxia against the closely related dengue virus was shown to increase the frequency of severe disease in dengue virus-naive children (55, 56). Thus, NS1 may be an overlooked component of a safe and effective ZIKV vaccine.

Recently, several groups have reported the effectiveness of NS1-based vaccines against ZIKV infection. In a paper by Brault et al., an NS1 vaccine incorporated into a modified vaccinia virus Ankara (MVA) vector was shown to protect wild-type mice against intracerebral challenge (40). As the same mice were vaccinated and subsequently challenged, it is unclear whether cell-mediated or humoral immunity or both contributed to protection. Studies by Liu et al. and Li et al. incorporated NS1 in addition to PrM/M and E in either an adenovirus 2 (Ad2)- and a recombinant vesicular stomatitis virus (rVSV)-based vaccine, respectively (41, 42). Both studies showed that the inclusion of NS1 into the vaccine construct provides additional protection compared to PrM/M and E alone. Though the contribution of NS1 antibodies to protection is clearly shown, an Ad2-NS1 construct alone was not tested. However, an rVSV-NS1 construct without any structural protein components was shown to reduce viral titers compared to those in the unvaccinated control mice.

Our study demonstrates that a vaccination strategy based solely on the ZIKV NS1 protein can elicit a strong antibody response that significantly protects mice against lethal challenge (Fig. 2 and 3). This strategy involved priming vaccinated mice with a DNA plasmid, followed by two protein boosts with either Freund’s adjuvant or AddaVax, which is an oil-in-water emulsion similar to MF59 found in a human seasonal influenza virus vaccine (57). We found that antibodies elicited by this vaccine bound potently to soluble NS1 protein by ELISA and recognized ZIKV-infected Vero cells, as measured by immunofluorescence. Pooled sera from vaccine groups also activated Fc-FcγR-mediated effector functions against infected Vero cells or NS1-transfected 293T cells in an ADCC reporter assay.

In contrast to previous work, our study used a passive-transfer model in which sera from vaccinated mice were passively transferred to STAT2^−/−^ mice. The mice then underwent a lethal challenge via intradermal infection of two ZIKV strains from different lineages (Fig. 3). Though the ZIKV sequences are highly conserved, the two different strains were isolated 68 years apart and display different disease phenotypes in STAT2^−/−^ mice (45). We first tested the efficacy of our NS1 vaccination strategy against the ZIKV MR766 strain, which due to its high lethality in mice represents a stringent challenge. The high lethality of MR766 was likely due to extensive passaging in the brains of mice. In this case, four of five mice receiving serum from the NS1 Freund’s adjuvant group and three of five mice from the NS1 AddaVax group survived. In contrast, none of the mice receiving serum from control vaccinated mice survived infection. The ZIKV PRVABC59 strain isolated in 2015 represents the modern circulating strain, was not mouse adapted, and is less pathogenic in mice. In this challenge model, all mice receiving serum from NS1-vaccinated mice survived, while two of four mice receiving serum from control-vaccinated mice succumbed to infection. Notably, none of the vaccinated mice were completely protected from ZIKV disease, as measured by weight loss or clinical score, suggesting that sterilizing immunity is not achieved. However, this is the first demonstration of NS1 antisera providing protection against lethal ZIKV challenge in a passive-transfer model, underscoring the importance of NS1-specific antibodies in mediating immunity to ZIKV. Though the exact mechanism of NS1-specific immunity needs to be further studied, we speculate that Fc-mediated viral clearance plays an important role in the prevention of disease progression by clearing virus-infected cells. Furthermore, both the AddaVax and Freund’s adjuvant treatment groups elicit high titers of antibodies and protection in passive-transfer studies that do not significantly differ from each other. Therefore, we are unable to quantify a minimally protective NS1-specific titer. Future studies might perform dose titrations of vaccine to determine the minimal NS1-specific antibody titer required for protection.

To determine whether NS1-mediated immunity is relevant and long-lived in humans, we obtained 31 serum samples from 16 different patients who were infected by ZIKV. These samples ranged from day 3 to day 267 postonset of symptoms and represent both the acute and the convalescent phase of illness. We tested binding to recombinant NS1 by ELISA and found that the antibodies become detectible by day 10 and last beyond day 267 (Fig. 4). Based on our results, we conclude that the NS1 protein of ZIKV is potently immunogenic. These results are to be expected, as the NS1 response in other flaviviruses has been well studied. To determine whether antibodies elicited by natural infection were functionally active, we performed an assay to measure Fc-mediated effector functions. All serum samples that were positive by ELISA were also positive in the reporter assay. In contrast, negative-control sera and sera from day 3 postonset of symptoms were unable to elicit Fc-mediated effector functions on infected cells. Additionally, all serum samples that were active against infected Vero cells were also active against NS1-transfected 293T cells (Fig. 5). This suggests that the predominant Fc-mediated antibody response against Zika virus targets the NS1 protein. Additionally, the NS1 protein was shown to be sufficient to activate Fc-mediated effector functions on infected cells by human sera. We have previously shown human monoclonal antibodies that target ZIKV NS1 are protective. However, future studies will look at purified polyclonal NS1 antibodies isolated from human sera to determine if passive transfer of these antibodies will protect mice against lethal challenge.

Fc-dependent responses mediated by virus-specific antibodies can generally be divided into two categories: responses that target viral particles and responses that mediate killing of virus-infected cells. In the first scenario, antibodies can facilitate the internalization of virions via Fc-mediated endocytosis into innate immune cells, where either degradation or replication can occur (58, 59). In the context of Zika virus and other flaviviruses, antibody-mediated uptake of virus increases the sites of virus replication and can potentially enhance disease (18). Alternatively, antibodies can direct the killing of virus-infected cells by activating innate immune cells, such as natural killer cells, macrophages, and neutrophils, via Fc-FcγR interactions (60). Here, we wanted to ask whether envelope-specific antibodies can facilitate similar levels of Fc-mediated clearance of virus-infected cells.

In contrast to the NS1 protein, the ZIKV envelope protein is not expressed at the cell surface (15). Nascent flaviviral particles bud internally from the Golgi apparatus, and structural proteins are not readily accessible on the surfaces of infected cells. Therefore, we hypothesized that while envelope-specific antibodies can bind intact virion to elicit ADE, these antibodies are unable to bind infected cells and, thus, cannot evoke protective Fc-mediated effector functions as measured by our *in vitro* reporter assay. Here, we demonstrate that TBEV-vaccinated individuals can elicit cross-reactive antibodies toward the Zika virus E protein. The cross-reactivity of antibodies between TBEV vaccinees and ZIKV is not surprising given that many flaviviruses have common epitopes on the surface of the E protein. The TBEV vaccine preparation uses inactivated TBEV virus. Therefore, the NS1 component is not part of the TBEV vaccine, and there is no measurable antibody response to the ZIKV NS1 protein. As we expected, these cross-reactive envelope antibodies could not engage FcγRs in our reporter assay when tested on virus-infected cells (Fig. 6). ADE of infection occurs when measured *in vitro*, however, because virions prominently display conserved fusion loop epitopes contributing to enhanced viral uptake. We hypothesize that while E-specific antibodies are superior in providing sterilizing immunity against Zika virus infection, their protective efficacy can be limited by the possibility of ADE. Additionally, E-specific antibodies are not efficient in the clearance of virus-infected cells because of the lack of envelope protein displayed at the cell surface. In contrast, NS1-specific antibodies can direct the clearance of virally infected cells, as shown previously (32).

Overall, our work further establishes the importance of NS1 as a component of a safe and effective Zika virus vaccine. Our data may explain how the incorporation of an NS1 component can enhance the effectiveness of a candidate ZIKV vaccine containing structural components only (42). We hypothesize that the design of a safe and effective ZIKV vaccine will benefit from the incorporation of an NS1 immunogen to induce potent Fc-mediated immunity that clears virus-infected cells. We show that antibodies elicited by an NS1-based vaccine can protect in a lethal-challenge model and are functionally active as measured by a surrogate ADCC assay. Furthermore, NS1-specific antibodies are robust and long-lasting in humans and, based on our mouse experiments, can provide protection against ZIKV disease via Fc-FcγR interactions.

## MATERIALS AND METHODS

### Cells and viruses.

Human embryonic kidney 293T (HEK 293T) cells (American Type Culture Collection [ATCC] catalog number CRL-1573) and African green monkey kidney (Vero) cells (ATCC) were grown in Dulbecco’s modified Eagle medium (DMEM; Gibco) supplemented with 10% fetal bovine serum (FBS) (HyClone) and antibiotics (100 units/ml penicillin-100 µg/ml streptomycin [Pen-Strep]; Gibco). Human embryonic kidney Expi293F cells (Gibco) were grown in Expi293 expression media. The ZIKV PRVABC59 virus (2015/Puerto Rico, BEI NR-50684) and ZIKV MR766 virus (Rhesus/1947/Uganda, BEI NR-50065) were obtained from BEI Resources. Zika viruses were propagated in Vero cells in 1× minimum essential medium (MEM); after 72 h postinfection (hpi), cell culture supernatants were harvested, aliquoted, and stored at −80°C until use.

### Recombinant Zika virus NS1.

Two mammalian expression plasmids expressing NS1 of ZIKV PRVABC59 (2015/Puerto Rico; GenBank accession number KU501215) were generated by incorporating the last 24 amino acids of ZIKV envelope (NGSISLMCLALGGVLIFLSTAVSA) to the amino terminus of the NS1 coding region; the entire sequence was human-codon optimized using the Integrated DNA Technologies Codon Optimization tool. The first construct contained only the partial envelope and whole NS1 coding regions by inserting the synthetic gene insert into pCAGGS digested with NotI and XhoI (New England Biosciences), resulting in pCAGGS NS1 (Fig. 1A). Another construct, a PreScission Protease cleavage site (LEVLFNGPG) and a hexahistidine motif (HHHHHH) were added to the carboxy terminus of the NS1 coding region, resulting in pCAGGS NS1-His (Fig. 1B). Both constructs were generated using homologous recombination (In-Fusion; TaKaRa). To generate recombinant NS1 proteins, 30 ml of Expi293 cells were transfected with 30 µg of pCAGGS-NS1-His plasmids and 81 µl of ExpiFectamine transfection reagent (Gibco) as per the manufacturer’s instructions. After 120 h, cells were pelleted by low-speed centrifugation and sonicated. Sonicated cells were pelleted again by centrifugation, and the supernatant was removed and incubated with Ni-NTA resin overnight at 4°C. The resin-supernatant mixture was then passed over 10-ml polypropylene columns (Qiagen). The retained resin was washed four times with 15 ml of washing buffer (50 mM Na_2_HCO_3_, 300 mM NaCl, 20 mM imidazole, pH 8), and protein was eluted with elution buffer (50 mM Na_2_HCO_3_, 300 mM NaCl, 300 mM imidazole, pH 8). The eluate was concentrated using Amicon Ultracel (Millipore) centrifugation units with a cutoff of 10 kDa, and buffer was exchanged with phosphate-buffered saline (PBS) at pH 7.4. Protein concentration was quantified using a Pierce bicinchoninic acid protein assay kit (Thermo Scientific) with a BSA standard curve. Purified soluble NS1 proteins were resolved in a reducing and denatured SDS-PAGE gel (in monomeric forms of around 45 kDa and in homodimeric forms of around 90 kDa) and visualized using SimplyBlue SafeStain (ThermoFisher, Inc.).

### ELISA.

Immulon 4 HBX ELISA plates (Thermo Scientific) were coated with recombinant ZIKV PRVABC59 NS1 protein (produced in-house) or recombinant envelope protein (MyBioSource accession number MBS319787) at 2 μg/ml in pH 9.41 carbonate buffer overnight at 4°C. Plates were washed three times with PBS between each step. After being blocked with 5% nonfat (NF) milk for 1 h, mouse sera were incubated at a starting concentration of 1:50, serially diluted 4-fold, and incubated for 2 h at room temperature. For experiments using human sera, a starting concentration of 1:40 was used. Horseradish peroxidase (HRP)-conjugated goat anti-human IgG antibody (AP504P; Millipore Sigma) or anti-mouse IgG antibody (AP503P; Millipore Sigma) was used to detect binding of IgG antibodies, followed by development with the HRP substrate (SigmaFast OPD; Sigma-Aldrich). Reactions were stopped by the addition of 3 M HCl, and absorbance was measured at 490 nm on a microplate spectrophotometer (Bio-Rad). Experiments were performed in duplicate. A nonparametric multiple-comparison Kruskal-Wallis test was utilized to examine significance between groups. GraphPad Prism 6 was used to calculate area under the curve (AUC) values.

### Immunofluorescence.

A 24-well plate was treated with 30 µg/ml of poly-d-lysine (Millipore) for 1 h, followed by three washes with 1× PBS and a final wash with complete cell culture medium. HEK 293T cells (2 × 10^5^ cells/well) were transfected in suspension with 0.5 µg of plasmid DNA (pCAGGS NS1 or pCAGGS NS1-His) and 2 µl of Lipofectamine 2000 (Invitrogen). Twenty-four hours posttransfection, cells were fixed with 0.5% paraformaldehyde (PFA)-1× PBS for 30 min. Cells were blocked with 5% nonfat milk for 30 min at room temperature, followed with incubation of 10 µg/ml of a human monoclonal antibody AA12 (32) or rabbit polyclonal antihistidine antibody (ThermoFisher) diluted in 1% nonfat milk-1× PBS for 1 h. Anti-human or anti-rabbit antibody conjugated to Alex Fluor 488 (Invitrogen) diluted in 1% nonfat milk-1× PBS at 1:1,000 were used as secondary antibodies. Stained cells were visualized using a Celigo imaging cytometer. Vero cells were infected with Zika virus PRVABC59 at a multiplicity of infection (MOI) of 0.5. After 24 h postinfection, the monolayer of Vero cells was fixed with 0.5% PFA-1× PBS. Cells were blocked with 5% nonfat milk for 30 min at room temperature. Blocking buffer was then discarded, and sera were added at a dilution of 1:100 in nonfat milk for 2 h at room temperature. Plates were washed three times with PBS between each step. After the cells were washed, an anti-mouse IgG secondary antibody conjugated to Alexa Fluor 488 (ThermoFisher) diluted 1:500 in nonfat milk was added to the monolayer, and plates were incubated in the dark for 1 h at room temperature. The cells were washed with PBS, and the monolayer was visualized using an Advanced Microscopy Group (AMG) Evos microscope (ThermoFisher).

### Antibody-dependent effector functions.

For experiments involving infected cells, Vero cells were seeded on 96-well, flat, white-bottom plates (Corning) and infected after 24 h with Zika virus PRVABC59 at an MOI of 0.01. For experiments involving transfected cells, HEK 293T cells were seeded onto 96-well, poly-d-lysine-coated, flat, white-bottom plates (Corning). After 24 h, the cells were transfected with 100 ng per well of pCAGGS-NS1 without the hexahistidine tag. At 16 h posttransfection or 40 h postinfection, the medium was removed and 25 μl of assay buffer (RPMI 1640 with 4% low-IgG FBS) was added to each well. Then, sera were added in a volume of 25 μl at a starting dilution of 1:75 and serially diluted 3-fold in assay buffer in duplicate. The sera were then incubated with the transfected or infected cells for 30 min at 37°C. Genetically modified Jurkat cells expressing either mouse FcγR IV or human FcγR IIIa with a luciferase reporter gene under the transcriptional control of nuclear-factor-activated T cell (NFAT) promoter were added at 7.5 × 10^4^ cells in 25 μl per well, which is approximately a 1:2 ratio of target cells to effector cells (Promega). Cells were then incubated for another 6 h at 37°C. Bio-Glo Luciferase assay reagent was added, and luminescence was quantified using a microplate reader. Fold induction was measured in relative light units and calculated by subtracting the background signal from wells without effector cells and then dividing values for wells with antibody by values for those with no antibody added. Specifically, fold induction was calculated as follows: (RLU_induced_ – RLU_background_)/(RLU_uninduced_ – RLU_background_). The mean values and standard errors of the means (SEM) were reported, and a nonlinear regression curve was generated using GraphPad Prism 6.

### Mouse vaccination.

All animal experiments were performed in an animal biosafety level 2 plus facility in accordance with the Icahn School of Medicine at Mount Sinai Institutional Animal Care and Use Committees (IACUC). Groups of 10 female STAT2^−/−^ mice were vaccinated with 80 μg pCAGGS NS1-His, pCAGGS-NS1, or an empty vector in 40 µl of double-distilled H_2_O. DNA vaccines were delivered via intramuscular electroporation in the left posterior thigh muscles via a TriGrid electroporation device (Ichor Medical Systems). Protein-based vaccines (recombinant NS1 protein from PRVABC59 or BSA) were administered at a dose of 5 μg/mouse adjuvanted with either AddaVax (InvivoGen) intramuscularly at days 21 and 42 or Freund’s complete adjuvant subcutaneously at day 21 (Sigma-Aldrich) and Freund’s incomplete adjuvant subcutaneously at day 42 (Sigma-Aldrich). Six weeks after the last vaccination (day 84), animals were anesthetized with a ketamine-xylazine cocktail (0.15 mg of ketamine/kg of body weight and 0.03 mg of xylazine/kg per mouse), and serum was obtained via cardiac vein puncture.

### Passive-transfer studies.

Groups of 4 to 5 male and female B6.129-STAT2^−/−^ mice (kindly provided by Christian Schindler; Columbia University) were passively transferred intraperitoneally with 200 μl pooled sera from vaccinated mice. Control mice received 200 μl pooled sera from mice vaccinated with DNA with an empty vector and BSA. Mice were challenged intradermally with 1,000 PFU of Zika virus PRVABC59 or 10 LD_50_s Zika (158 PFU) virus MR766 and evaluated for 14 days. Mice were monitored daily for weight and clinical signs. Clinical scoring was conducted using the predefined criteria, with a maximum possible score of 7: impact on walking, unresponsiveness, left hind leg paralyzed, right hind leg paralyzed, left front leg paralyzed, and right front leg paralyzed. Deceased animals were given a score of 7. Animals that showed more than 25% weight loss or full paralysis were humanely euthanized. Experiments were conducted with a balanced amount of male and female mice and with an even distribution of mice from different litters whenever possible. To determine statistical significance, the Mantel-Cox and Gehan-Breslow-Wilcoxon tests were used for survival curves, and a multiple *t* test and the Holm-Sidak method were utilized to analyze the weight curve and clinical scores. An asterisk(s) on a graph indicates the statistical significance (*, *P* < 0.05) of a treatment group compared to the control group.

### Donor samples.

Deidentified TBEV-vaccinated donor serum samples were provided by the Vienna Blood Center in Austria as described previously (49). Deidentified Zika virus-infected blood donor plasma samples were obtained through the Global Virus Network Zika Serum Bank or Biodefense and Emerging Infections Research Resources Repository (BEI Resources).

### Study approval.

All studies conducted were considered by the Icahn School of Medicine at Mount Sinai’s Institutional Review Board as not human subject research (NHSR).

### Statistical analysis.

Results from multiple experiments are presented as means ± SEM. Multiple *t* tests were used to test for statistical differences between mean values. Data were analyzed with GraphPad Prism 6 software, and *P* values of <0.05 were considered statistically significant.
