# Supplementary material for: Antibodies Elicited by an NS1-Based Vaccine Protect Mice against Zika Virus
Source: mBio. 2019 Apr 2;10(2):e02861-18. doi: 10.1128/mBio.02861-18 (PMC6445944; doi:10.1128/mBio.02861-18)
Supplement: FIG S1 [file mBio.02861-18-sf001.docx]

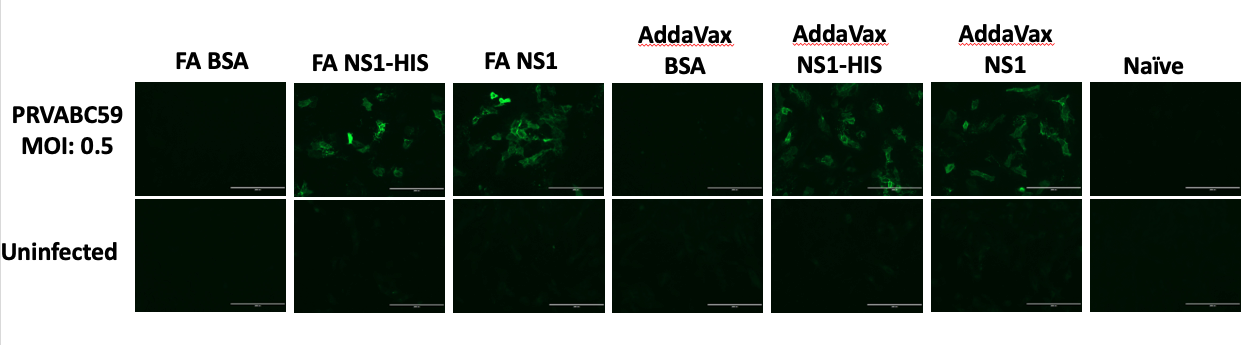


**Figure S1. Immunofluorescence of pooled mouse serum.**

Vero cells were infected with PRVABC59 ZIKV at an MOI of 0.5 for 24 hours. The cells were fixed with 0.5% paraformaldehyde and blocked with 5% non-fat milk. Serum were added at a dilution of 1:100 and an anti-mouse antibody conjugated to Alexa Fluor 488 was used as a secondary antibody. Scale bars are equal to 200 microns.
