# Supplementary material for: Antibodies Elicited by an NS1-Based Vaccine Protect Mice against Zika Virus
Source: mBio. 2019 Apr 2;10(2):e02861-18. doi: 10.1128/mBio.02861-18 (PMC6445944; doi:10.1128/mBio.02861-18)
Supplement: TABLE S1 [file mBio.02861-18-st001.docx]

| **Patient code** | **Age** | **Gender** | **Country of exposure** | **Days post illness onset** |
| --- | --- | --- | --- | --- |
| **UTMB1** | **n/a** | **F** | **Dominican Republic** | **22** |
| **UTMB1** | **n/a** | **F** | **Dominican Republic** | **58** |
| **UTMB2** | **32** | **F** | **Honduras** | **3** |
| **UTMB2** | **32** | **F** | **Honduras** | **7** |
| **UTMB2** | **32** | **F** | **Honduras** | **14** |
| **UTMB2** | **32** | **F** | **Honduras** | **28** |
| **UTMB2** | **32** | **F** | **Honduras** | **45** |
| **UTMB4** | **26** | **F** | **Caribbean Islands** | **18** |
| **UTMB5** | **28** | **F** | **Jamaica** | **25** |
| **UTMB5** | **29** | **F** | **Jamaica** | **32** |
| **UTMB7** | **15** | **F** | **Colombia** | **129** |
| **UTMB8** | **28** | **F** | **Colombia** | **113** |
| **UTMB9** | **43** | **M** | **El Salvador, Guatemala** | **145** |
| **UTMB10** | **33** | **M** | **Haiti** | **41** |
| **UTMB10** | **33** | **M** | **Haiti** | **97** |
| **UTMB11** | **n/a** | **n/a** | **Haiti** | **56** |

**Table S1. Serum samples obtained from Zika virus infected patients.**

Serum samples obtained via the Global Virus Network. Patient code, age, gender, country of exposure and date of illness onset are shown.
