# Supplementary material for: Antibodies Elicited by an NS1-Based Vaccine Protect Mice against Zika Virus
Source: mBio. 2019 Apr 2;10(2):e02861-18. doi: 10.1128/mBio.02861-18 (PMC6445944; doi:10.1128/mBio.02861-18)
Supplement: TABLE S2 [file mBio.02861-18-st002.docx]

| **Patient code** | **Days post illness onset** |
| --- | --- |
| **NR-50611** | **233** |
| **NR-50613** | **236** |
| **NR-50615** | **224** |
| **NR-50617** | **267** |
| **NR-50619** | **202** |
| **NR-50621** | **202** |
| **NR-51058** | **6** |
| **NR-51079** | **6** |
| **NR-51118** | **7** |
| **NR-50808** | **“Acute”** |
| **NR-50809** | **“Acute”** |
| **NR-50810** | **“Acute”** |
| **NR-50818** | **“Acute”** |
| **NR-50819** | **“Acute”** |
| **NR-50820** | **“Acute”** |

**Table S2. Serum samples obtained from Zika virus infected patients.**

Serum samples obtained via BEI resources. Patient code and date of illness onset are shown.
